# Use of Cis-[^18^F]Fluoro-Proline for Assessment of Exercise-Related Collagen Synthesis in Musculoskeletal Connective Tissue

**DOI:** 10.1371/journal.pone.0016678

**Published:** 2011-02-11

**Authors:** Dorthe Skovgaard, Andreas Kjaer, Katja Maria Heinemeier, Malene Brandt-Larsen, Jacob Madsen, Michael Kjaer

**Affiliations:** 1 Institute of Sports Medicine Copenhagen, Bispebjerg Hospital and Faculty of Health Sciences, University of Copenhagen, Copenhagen, Denmark; 2 Cluster of Molecular Imaging, Faculty of Health Sciences, University of Copenhagen, Copenhagen, Denmark; 3 Department of Clinical Physiology, Nuclear Medicine and PET, Rigshospitalet, Copenhagen, Denmark; University of Texas, M.D. Anderson Cancer Center, United States of America

## Abstract

Protein turnover in collagen rich tissue is influenced by exercise, but can only with difficulty be studied *in vivo* due to use of invasive procedure. The present study was done to investigate the possibility of applying the PET-tracer, *cis-*[^18^F]fluoro-proline (*cis*-Fpro), for *non-invasive* assessment of collagen synthesis in rat musculoskeletal tissues at rest and following short-term (3 days) treadmill running. Musculoskeletal collagen synthesis was studied in rats at rest and 24 h post-exercise. At each session, rats were PET scanned at two time points following injection of *cis*-FPro: (60 and 240 min p.i). SUV were calculated for Achilles tendon, calf muscle and tibial bone. The PET-derived results were compared to mRNA expression of collagen type I and III. Tibial bone had the highest SUV that increased significantly (p<0.001) from the early (60 min) to the late (240 min) PET scan, while SUV in tendon and muscle decreased (p<0.001). Exercise had no influence on SUV, which was contradicted by an increased gene expression of collagen type I and III in muscle and tendon. The clearly, visible uptake of cis-Fpro in the collagen-rich musculoskeletal tissues is promising for multi-tissue studies *in vivo*. The tissue-specific differences with the highest basal uptake in bone are in accordance with earlier studies relying on tissue incorporation of isotopic-labelled proline. A possible explanation of the failure to demonstrate enhanced collagen synthesis following exercise, despite augmented collagen type I and III transcription, is that SUV calculations are not sensitive enough to detect minor changes in collagen synthesis. Further studies including kinetic compartment modeling must be performed to establish whether *cis*-Fpro can be used for non-invasive *in-vivo* assessment of exercise-induced changes in musculoskeletal collagen synthesis.

## Introduction

Collagen is the major structural protein in the extracellular matrix of the musculoskeletal tissues and constitutes approximately 90% of the total protein of tendons and bones as well as a substantial part of the connective tissue in skeletal muscles [1]–[2]. There is growing evidence that the musculoskeletal connective tissues can adapt to acute exercise more dynamically than hitherto thought and that both acute and more long-term exercise regimes result in an increase in collagen synthesis [3]–[7]. However, *in-vivo* investigations of these exercise-induced modifications rely on tissue invasive procedures with either placement of microdialysis fibers into or biopsies obtained from the studied tissues. A method for studying collagen synthesis in the musculoskeletal tissues *in vivo* without the use of invasive procedures would be advantageous in animal and human exercise models, both with regards to limiting the invasiveness and because such a method would allow for easier investigation of several collagen rich tissues, e.g. tendon, ligament and bone, simultaneously.

The amino acid, *proline* is a major constituent of collagen and is therefore widely used as a marker of collagen synthesis by measuring incorporation of either radioactive (in animals) or non-radioactive isotopic-labeled proline (^3^H-proline, ^14^C-proline, ^13^C-proline, ^15^N-proline) directly in the investigated tissues [1], [6], [8]–[11]. The labeled amino acid *cis-*[^18^F]fluoro-proline (*cis*-Fpro) was developed some years ago as a tracer for non-invasive, Positron Emission Tomography-based assessment of collagen synthesis [12]. The initial studies showed promising results with *cis-*Fpro detecting fibrosis in a rabbit model of silicose-induced pulmonary fibrosis [13]–[14], and in tumor-bearing mice with accumulation in osteosarcomas, mammary and colon carcinomas where a significant protein incorporation into collagen-rich tissue was found within 4 hours [12]. However, recently other studies have questioned the usefulness of *cis*-Fpro for detection of collagen synthesis [15]–[17].

Importantly, *cis*-Fpro has until now only been used for investigating collagen synthesis in relation to pathologically fibrotic processes. In contrast, the present study was designed to investigate the possibility of applying this particular PET tracer, *cis*-Fpro, for non-invasive assessment of collagen synthesis in musculoskeletal tissues of rats both in the resting state and after they have been subjected to a short-term (3 days) treadmill exercise regime. Furthermore, we aimed to validate a possible increase in the image-derived values for collagen synthesis in skeletal muscle and tendon by investigating the gene expression of the two major types of collagen; Collagen type I and Collagen type III using real-time PCR.

## Materials and Methods

### Animals

Male Wistar rats weighing 300 g were obtained from Charles River (Germany). The rats were housed in pairs and fed chow and water ad libitum. The animal room was maintained at 21°C with a 12∶12-h light-dark cycle. The study was approved by the Animal Research Committee of the Danish Ministry of Justice. Approval ID: 2006-561-1124.

### Exercise

The rats were randomly divided into two groups: The Exercise group (n = 8) and the Control group (n = 12). The Exercise group was subjected to three days of running on a costum made motorized rat treadmill with increasing speed, length and duration of the exercise. A grid at the trailing end of the treadmill administered a mild shock encouraging the rats to run in avoidance. On the first day the speed of the treadmill was initially set to 10 m/min for familiarization and gradually increased to 17.5 m/min. All rats completed 20 minutes of running exercise at a total length of 230 m. The second day the rats ran 40 minutes (890 m) at a maximal speed of 25 m/min. The third day the rats completed an exhaustive run consisting of 60 minutes (1,250 m) at a maximal speed of 25 m/min. The Control group was not subjected to treadmill running but maintained normal cage activity.

### Experimental protocol

The rats in the Exercise group were PET scanned three days prior to starting the running regime and then again 24 hours after the last day of the training protocol. The Control group was equally scanned twice with one week in between, however, the rats in this group sustained normal cage activity at all times. Before PET/CT scans the rats were anaesthetized with a subcutaneous injection of Hypnorm/Dormicum (5 mg/0.625 mL/kg), that was repeated (2.5 mg/0.313 mL/kg) every 20 min through a subcutaneous catheter during the experiment in order to maintain the anaesthesia. An iv catheter (BD Neoflon™ 24GA) for tracer injection was placed in a lateral tailvein. The tracer, *Cis*-Fpro, was given as a bolus and an uptake period of 60 minutes was allowed before the first PET scanning was performed. This first PET supposedly reflects proline transport [12]. An additional late PET scan was performed 240 minutes after tracer injection representing incorporation of the tracer into protein [12]. Before the first and between the two PET scans the rats stayed in their cages. Prior to each scan the rats were placed in a prone position on the acquisition bed with a heating pad (M2M, Cleveland, USA) attached. PET/CT scans were performed with the two hindlimbs in the field of view. In both groups the animals were sacrificed immediately after the last PET scan and both the Achilles tendons and soleus muscles were carefully cut out. Either right or left Achilles tendon and soleus muscle (randomly chosen) was immediately used for assessment of the protein-bound activity by protein precipitation with tricholoroacetic acid. The opposite Achilles tendon and soleus muscle were placed in RNAlater for subsequent analysis of gene expression of Collagen type I (COL1A1) and Collagen type III (COL 3A1). The radioactivity was immediately measured in these tissue samples using a γ-counter (Cobra II, model 5003, Packard, Meriden, CT; USA). Samples were kept in RNAlater overnight; whereafter RNAlater was removed and the samples were stored at −80°C for later use.

### Cis-Fproline synthesis

*N*-Boc-*trans*-4-tosyloxy-L-proline methyl ester was purchased from Advanced Biochemical Compounds (ABX). [^18^O]water was purchased from Rotem Industries. Kryptofix 222 (4,7,13,16,21,24-hexaoxa-1,10-diazabicyclo[8.8.8]-hexacosane) was purchased from Acros Organics. K_2_CO_3_ was purchased from UniKem. MeOH was purchased from Applichem. MeCN was purchased from Sigma-Aldrich. TFMSA (trifluoromethanesulfonic acid) was purchased from Fluka. KHCO_3_ and Na_3_PO_4_ were purchased from Merck. SepPak QMA light was purchased from Waters. SCX Isolute 1 g 6 ml column was purchased from Biotage.

Preparative HPLC was performed using a Phenomenex Luna 5 µm NH_2_ 100 A column (10×250 mm) on a Knauer K120 HPLC pump and UV detector connected to an inline radiodetector. Mobile phase: 75% MeCN, 25% N(CH_3_)_3_H_3_PO_4_, flow rate 7 ml/min. 4-[^18^F]fluoro-*trans*-proline: t_R_ = 8.5 min, 4-[^18^F]fluoro-*cis*-proline: t_R_ = 12.5 min.

Analytical HPLC was performed using a Waters Spherisorb 5 µm NH_2_ column (4.6×150 mm) on a Gilson HPLC pump and UV detector connected to an inline radiodetector. Mobile phase: 75% MeCN, 25% N(CH_3_)_3_H_3_PO_4_. Flow rate 1 ml/min. 4-[^18^F]fluoro-*trans*-proline: t_R_ = 5.7 min, 4-[^18^F]fluoro-*cis*-proline: t_R_ = 8.3 min.

No-carrier-added aqueous [^18^F]fluoride ion was produced via the [^18^O(p,n)^18^F] nuclear reaction by irradiation of a 2.4 ml [^18^O]water (98%-enriched) target on a CTI Eclipse (11 MeV proton beam) or on a Scanditronix MC32 (16 MeV proton beam).

The radiosynthesis of 4-[^18^F]fluoro-*cis*-proline was performed in accordance to a previously published procedure [18] with minor modifications. In short:

The aqueous [^18^F]fluoride was trapped on a QMA column, eluted with 1 ml Kryptofix222 solution (5.4 mg potassium carbonate, 20 mg Kryptofix 222, 500 µL H_2_O, 500 µL MeOH), and transferred to a sealed vial. The kryptofix solution was evaporated at 85°C and coevaporated twice with 1 ml MeCN.

*N*-Boc-*trans*-4-tosyloxy-L-proline methyl ester (16 mg) was dissolved in 1 ml anhydrous MeCN, added to the reaction vial, and reacted with [^18^F]fluoride for 10 minutes. MeCN was evaporated, and 0.5 ml 2 M TFMSA was added. The solution was heated in a closed vessel to 127°C and left for 10 minutes. The solution was cooled to room temperature and neutralized with 0.5 ml sat. KHCO_3_. 4 ml EtOH was added, and 4-[^18^F]fluoro-*trans*-proline was separated from 4-[^18^F]fluoro-*cis*-proline by preparative HPLC. The trans-cis ratio was typically 7/93 before purification.

The collected fraction was purified on a SCX column (preconditioned with 5 ml EtOH and 10 ml H_2_O). The column was washed with 10 ml H_2_O, and 4-[^18^F]fluoro-*cis*-proline was eluted with 6 ml 0.1 M Na_3_PO_4_ in 16±8% (n = 5) overall radiochemical yield. The radiochemical purity was 93–99%.

### PET/CT

CT data were acquired with a MicroCAT™ II Tomograph (Siemens Medical Solutions). The x-ray tube was set at 40 kVp and had 0.5 mm plate of added aluminium filtration. Exposure time was 700 ms per projection and the tube current was 500 µA. A total of 360 projections were used for a full 360° scan. Images were reconstructed using the Sheep-Logan algorithm. These settings had in pilot studies shown to provide clear images of the soft tissues (tendon and muscle).

PET data were acquired with a microPET® Focus™ 220 (Siemens Medical solutions, Malvern, PA, USA). Static emission scan (20 minutes) was acquired with the rats placed in a prone position on the acquisition bed and the two hindlimbs in the field of view. All listmode data were sorted into 3D sinograms using a span of 3 and a ring difference of 47 and reconstructed using Ordered Subset Expectation Maximization 2D (OSEM2D). Images were attenuation corrected using the CT-based attenuation correction method and in addition corrected for dead time, decay time and the system was calibrated to provide the quantification unity Bq/cc.

The uptake of *cis*-Fpro in the Achilles tendon, the triceps surae muscle (covering both gastrocnemius and soleus) and tibial bone was quantitated (Bq/cc) from each animal using the image analysis software Inveon (Siemens Medical solutions). The software allows for fusion of the PET images with CT for anatomical information. The alignment of the two image modalities were done on the basis of three distinctive fiducial markers placed directly on the animal bed within the field of view although not in proximity of the regions of interest (ROIs) (≥5 mm). The 3D ROIs were drawn on the image from the CT scan intending to cover I) the whole Achilles tendon from the calcaneal insertion to the musculo-tendinous region, II) the midportion of the triceps surae muscles, III) trabecular bone at the proximal part of the tibia and IV) cortical bone consisting of a ROI covering the circumference of the midshaft of the tibia. ROIs were drawn on both hindlimbs and the values given as an average of two sides. During subsequent analysis Standardized Uptake Values (SUVs) in the individual ROIs were calculated by dividing the mean specific activity with injected dose and weight of the individual rat assuming (1 cm^3^ = 1 g).

### Protein incorporation

In both Achilles tendon and soleus muscle the protein-bound activity was determined by protein precipitation with trichloroacetic acid (TCA). Initially the tissue samples were placed in a buffer containing 0.15 M NaCl, 0.1% Triton X-100 and 0.02 M Tris-HCl pH 7.4 [6] and homogenized using Bertin Precellys 24 (Bertin Technologies, France) and ceramic beads (CK 28, Bertin Technologies, France) with a program consisting of 6,000 g for 20 seconds. This was repeated twice for soleus muscle, while for the Achilles tendon it was necessary to repeat the procedure 5 times in order to dissolve all visible tissue. Following homogenization 750 µl of the homogenate were transferred into a 1.5 mL Eppendorf and 250 µl TCA were added, subsequently the mixture was vortexed thoroughly and centrifuged 10 minutes at 4,000 g. Pellet and supernatant were divided, whereafter the procedure, with addition of 750 µl of TCA and centrifugation, was repeated. Upon the last centrifugation, the supernatant was removed and added to the first supernatant. The ^18^F-activity in both fractions was determined using a γ-counter. The percentage of protein incorporation was calculated as the amount of radioactivity in the pellet (protein fraction) related to the amount of radioactivity in the supernatant (non-protein fraction).

### Gene expression

Total RNA from all tissue types was extracted according to the method described by Chomzynski and Sacchi [19]. In brief, muscle and tendon tissue was homogenized in TRI-reagent (Molecular Research Center, Inc.) using Bertin Precellys 24 (Bertin Technologies, France) homogenizer with the aid of ceramic beads (CK 28, Bertin Technologies, France) and a program consisting of 6,000 g for 20 seconds. This was repeated twice for soleus muscle, while for the Achilles tendon it was necessary to repeat the procedure 5 times until all visible tissue was dissolved. Following homogenization, BCP (1-bromo-3-chloropropane) (MRC) was added (100 µL per 1000 µL TRI-reagent) in order to separate the samples into an aqueous and an organic phase. From the aqueous phase, RNA was precipitated using isopropanol. The RNA pellet was washed in ethanol and subsequently dissolved in RNAse-free water. RNA integrities were determined by Bio-analyzer 2100 (Agilent Technologies, CA, USA) and concentration by spectrophotometry. Subsequently 300 ng total RNA from muscle and 200 ng total RNA from tendon were reverse transcribed using StrataScript® QPCR cDNA Synthesis Kit (Stratagene, La Jolla CA, USA). Upon this, samples were stored at −80°C until further analyzes. For each target mRNA, 0.25 µL cDNA was amplified in a 25 µL SYBR Green PCR reaction containing 1× Quantitect SYBR Green Master Mix (Qiagen, CA, USA) and 100 nM of each primer. The amplification was monitored real-time using the MX3000P real-time PCR machine (Stratagene, CA, USA). The threshold cycle (*C_t_*) values were related to a standard curve made with the cloned PCR products and specificity was confirmed by melting curve analysis after amplification. COL1A1 and COL3A1 were investigated as the two genes of interest and for internal control two supposedly constitutive genes RPL0 and GAPDH were chosen (table 1). However, as we have previously observed, the expression of these “housekeeping” genes were influenced by the intervention [3]–[4] with significant changes following strength training and hindlimb unloading in RPLP0/GAPDH ratio and GAPDH and RPLP0 mRNA relative to total RNA and relative to tissue weight. We therefore chose to express mRNA data relative to tissue weight as we have done previously. This choice of normalization is thoroughly discussed in earlier publications [3]–[4], [20]. Data for mRNA expression are presented as fold changes relative to the mean of the values in the control group.

**Table 1:** PCR Primers.

mRNA	Forward primer (5′-3′)	Reverse primer (5′-3′)
COL1A1	ATCAGCCCAAACCCCAAGGAGA	CGCAGGAAGGTCAGCTGGATAG
COL3A1	TGATGGGATCCAATGAGGGAGA	GAGTCTCATGGCCTTGCGTGTTT
GAPDH	CCATTCTTCCACCTTTGATGCT<1/emph>	TGTTGCTGTAGCCATATTCATTGT
RPL0	AGGGTCCTGGCTTTGTCTGTGG	AGCTGCAGGAGCAGCAGTGG

### Statistical analysis

Within each group *t test for paired samples* was used for comparing the SUV of *Cis*-Fpro in the pre-exercise PET and the post-exercise PET scan comparing both the early “*Cis*-Fpro-transport” (60 minutes post injection) and “*Cis*-Fpro-incorporation” (240 minutes post injection). For comparison of SUV of the aforementioned values between the Exercise and Control group *t test for unpaired samples* was used. Data for the two genes of interest COL1A1 and COL3A1 were log transformed before analyzes and compared between the two groups (Exercise vs. Control) with *t test for unpaired samples*. The statistical analyzes were carried out using SPSS 16.0. A P level of 0.05 was considered significant. The results are expressed as mean ± SEM, except gene expression data, which are presented as geometric means +/− backtransformed SEM.

## Results

The two groups were similar with respect to bodyweight on the day of the first PET scan (Exercise: 339±12 and Control 353±17 g) and both gained the same amount of weight during the week between the two PET scans (approximately 20 g). Furthermore, the rats received similar doses of the injected tracer (overall mean for the two groups at both PET sessions 33.1±1.87 MBq). The volume of the ROIs in tendon were 10.7±0.74 mm^2^, muscle 521.2±32.5 mm^2^, trabecular bone 10.2±0.73 mm^2^ and cortical bone 2,960±970 mm^2^.

### Uptake of Cis-Fpro in musculoskeletal tissue

**Figure 1:**
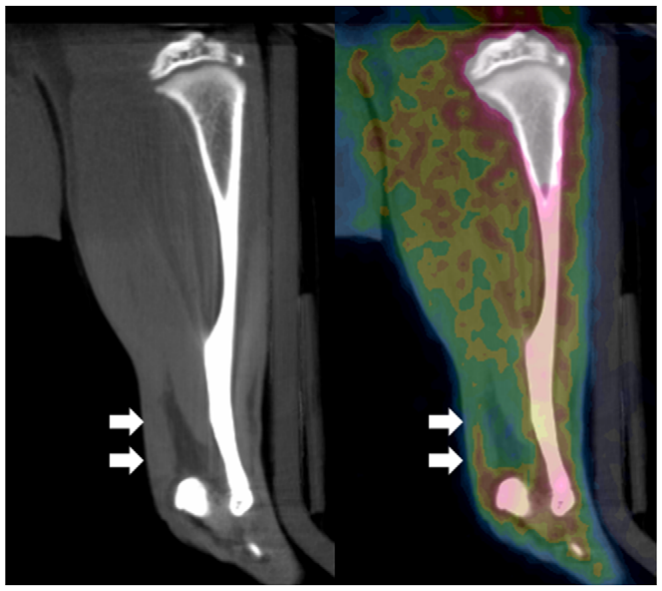
Representative sagittal CT/PET image of a rats hindlimb. A: CT sagittal plane showing the clearly distinctive Achilles tendon (white arrows). B: the corresponding *cis*-FPro-PET (superimposed on the CT image) reveals the tendon (white arrows) with a higher uptake of *cis*-FPro compared to adjacent anterior adipose tissue. The tibial bone has the highest uptake of *cis*-Fpro.

**Figure 2:**
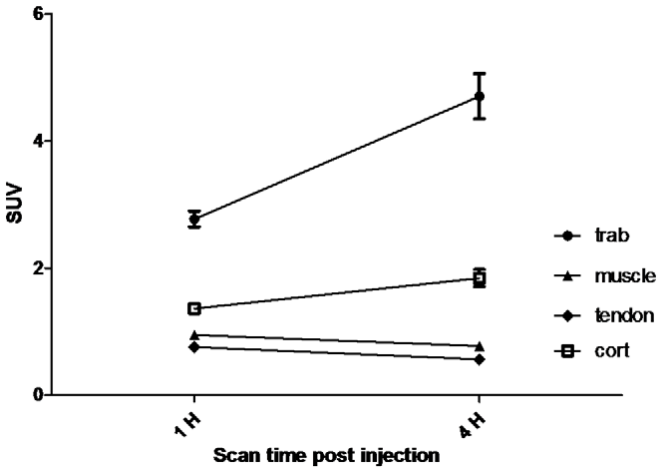
SUV of *cis*-FPro in musculoskeletal tissues 60 minutes and 240 minutes post-injection (resting levels). The figure illustrates the changes in *cis*-Fpro uptake in the musculoskeletal tissues at two time points following injection of the tracer. In trabecular and cortical bones *cis*-Fpro uptake increased significantly (p<0.001) when comparing the values from the early PET scan (60 minutes post injection) to the late (240 minutes post injection). This was contradicted by a continuous decrease in tendon and muscle (p<0.001). Values are the mean ± SEM of all rats in the Exercise (only pre-exercise values) and Control group at the two time points post injection.

Representative images of corresponding coronal CT and PET are shown in figure 1. The uptake of *cis*-Fpro was assessed one hour post-injection and 240 minutes post-injection. SUV of all the investigated tissues are presented in table 2. Trabecular and cortical bones (of both groups, Exercise and Control) revealed the highest values that increased significantly (p<0.001) when comparing the values from the early PET scan (60 minutes post injection) to the late (240 minutes post injection). This was in contrast to a continuous decrease in SUV in tendon and muscle (p<0.001) (figure 2).

**Table 2:** SUVs obtained in the two groups (Exercise and Control).

	60 minutes p.i. *Cis*-Fpro “transport”				240 minutes p.i. *Cis*-Fpro “incorporation”			
	control day 1	control day 8	pre-exercise	post-exercise	control day 1	control day 8	pre-exercise	post- exercise
tendon	0.75±0.01	0.76±0.01	0.77±0.02	0.74±0.03	0.58±0.06	0.57±0.01	0.56±0.02	0.54±0.03
muscle	0.95±0.02	0.97±0.02	0.97±0.02	0.91±0.05	0.72±0.05	0.80±0.02	0.78±0.03	0.79±0.06
cortical bone	1.46±0.04	1.40±0.04	1.34±0.03	1.27±0.10	1.91±0.05	1.91±0.10	1.73±0.12	1.83±0.27
trabecular bone	2.68±0.11	2.86±0.13	2.66±0.09	2.88±0.18	4.50±0.53	4.97±0.28	4.59±0.23	4.76±0.39

In the Exercise group *cis*-Fpro uptake was investigated pre exercise and 24 hours post exercise (three days of treadmill running). In each session the rats were PET scanned twice at two different time points following injection of *cis*-FPro: 60 minutes post-injection and 240 minutes post-injection. Each of the musculoskeletal tissues revealed a distinct uptake, however no changes were found following exercise. In the Control group *cis*-Fpro uptake was investigated twice with one week in between and revealed a constant uptake of *cis-*FPro comparable to that of the exercise group.

### Effect of exercise

**Figure 3:**
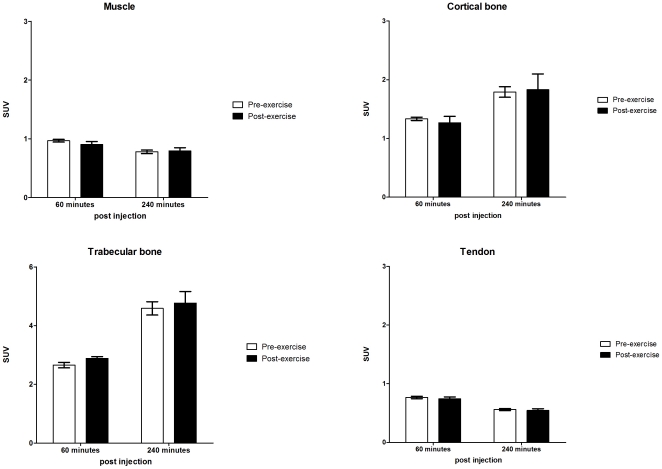
SUV of *Cis*-Fpro in the Exercise group in each of the musculoskeletal tissues. The SUV 60 minutes post injection did not change in any of the investigated tissues, when comparing pre- (open bars) and post-exercise (filled bars) PET scans. Likewise, no changes were found in the uptake of *cis*-Fpro 240 minutes post injection from the pre-exercise PET scan (open bars) to the post-exercise PETscan (filled bars). Values are mean ± SEM.

*Cis*-Fpro PET was obtained both before the three days of endurance exercise and 24 hours after the last exercise bout. Exercise did not change SUV in any of the investigated tissues, neither at one hour post injection (“*cis*-Fpro transport”), nor at four hours post injection (“*cis*-Fpro incorporation”) (figure 3). Likewise, SUV were constant in control group that were scanned twice interspersed with 8 days of normal cage activity. Furthermore, the image-derived results were confirmed by well counting on weighed tissue samples (based on tissues samples placed in RNAlater) from each rat. Also when relating counts/minute to tissue weight and injected dose no differences were found between the two groups (data not shown).

### Protein incorporation

Protein incorporation was only investigated following the very last PET scan (260 minutes post injection) and revealed that for skeletal muscle 20% (±0.1%) (n = 16) of the radioactivity was in the TCA precipitated protein fraction and in tendon 24% (±0.2%) (n = 11) was in the protein fraction. These values from the two investigated tissues were not significantly different and were not changed following exercise.

### COL1A1 and COL3A1 mRNA

**Figure 4:**
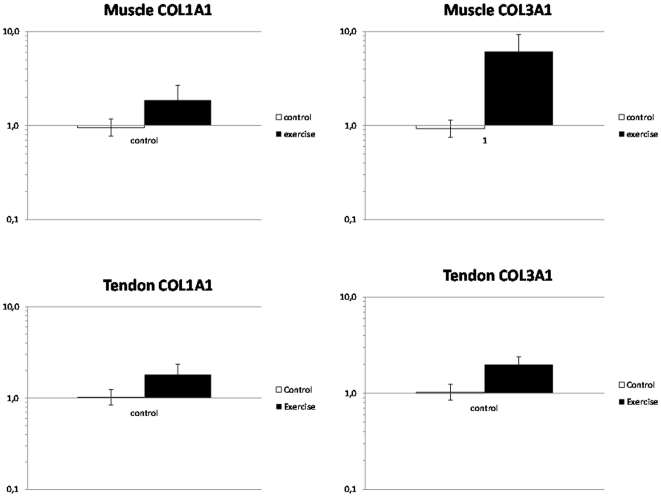
Gene expression of COL1A1 and COL3A1. COL1A1 and CoL3A1 mRNA normalized to tissue weight, presented as fold changes in the exercise group (filled bars) relative to the mean of the control group (open bars) in Achilles tendon and soleus muscle. In tendon COL3A1 mRNA was significantly increased (p<0.05) in the Exercise group and COL1A1 mRNA showed a strong tendency to an increase (p = 0.09). In exercised soleus muscle both COL1A1 (p<0.01) and COL3A1 (p<0.001) were significantly increased. Values are geometric means ± SEM.

The general range of *C_t_* values was 17–29. Compared to the control group, the soleus muscle of the exercise group exhibited a ∼2 fold increase of COL1A1 mRNA (p<0.01) and a ∼5 fold increase of COL3A1 mRNA (p<0.001). This was in Achilles tendon accompanied by a significant ∼2 fold increase of COL3A1 mRNA (p<0.05) and a strong tendency towards enhancement of COL1A mRNA (1.8 fold, p = 0.09) (figure 4).

## Discussion

The present study evaluated the possibility of using *cis-*[^18^F]fluoro-proline as a non-invasive method of assessing collagen synthesis in rat musculoskeletal tissues. With the use of PET it is possible to non-invasively study several regions and multiple tissues simultaneously. Importantly, the investigated tissues (tendon, muscle and bone) all revealed a visual uptake of the tracer (1 h and 4 h post-injection).

As expected the highest uptake of *cis*-Fpro was found in trabecular bone. This is in line with other tracer studies (with the use of ^13^C or ^15^N-proline) showing a higher basal collagen turnover in bone compared to that of other types of musculoskeletal tissues [1], [21]–[23] – with the trabecular bone as the most metabolically active part, possibly due to the extended surface to bone area [24]. In comparison, the uptake of *cis*-Fpro in calf muscle was higher than that of Achilles tendon, however, this is not necessarily reflecting a distinctively enhanced muscle-specific collagen turnover. In skeletal muscle collagen represents only 10–15% of the myofibrillar protein and in addition the collagen synthesis rate is lower than that of other muscle proteins [1], and therefore the PET-derived values for uptake of *cis*-Fpro in calf muscle most likely reflects a more unspecific amino acid transport and/or overall muscle protein synthesis since proline is a substrate for all proteins – although not as prevalent as in collagen molecules.

The tissue-specific difference in the kinetics of *cis*-Fpro following injection raises some questions. In bone the SUV of *cis*-Fpro increased from the early (60 minutes post injection) scan to the late (240 minutes post injection) scan. Opposed to this SUV of *cis*-Fpro in tendon and muscle decreased over time. This could indicate a tissue specific difference in the acceptance of *cis*-Fpro in collagen synthesis or even a hierarchical redistribution of the accessible *cis*-Fpro following the first initial uptake, with bone given the highest priority. However, another possible explanation is that ongoing metabolism of the *cis*-Fpro over time leads to defluorination of the proline, thereby releasing the bone-seeking fluorine-18. In the study by Wester et al. [12] a similar increased radioactivity in bone over time was found, but based on their results the authors argued against defluorination as an explanation, since a high in vivo instability of the *cis*-Fpro would result in a faster uptake of fluorine-18 into bone compared to tumor, which was not found. Furthermore, metabolic investigations in that study (HPLC and SEC of plasma and tissue homogenates) revealed no contribution of metabolites to the biodistribution and the activity eluted in the low molecular weight fraction was unchanged tracer.

Collagen turnover in animal and human tissues is rather complicated encompassing at least two pools of collagen, a soluble fast-turnover pool of procollagen/immature collagen monomers and a slower-turnover, insoluble pool of mature fibrillar collagen, which may be regulated independently [1]. The degradation of immature collagen has been found to be as fast as 15 minutes in rat tissues and a substantial part might even be degraded intra-cellularly, as observed from the appearance of ^14^C labeled hydroxyproline after application of labeled proline [25]. Therefore, the PET-based values of *cis*-Fpro uptake cannot be interpreted as an actual permanent tissue deposition of collagen.

Recently, several studies have seriously questioned the possibility of using *cis*-Fpro as a marker of collagen synthesis. In that context, *cis*-Fpro failed to map both scar formation in abdominal surgical mesh implants in rats [15], lungfibrosis [17] and tumors [16] in humans. In these studies especially the insecurity of the acceptance of the fluoro-labeled proline into protein was pointed out as a possible explanation and Zimny et al. [16] suggested that it might be worthwhile labeling proline with other radionuclides, possibly with longer halflife, since protein incorporation is a protracted process.

### Effect of exercise

SUV did not change in any of the studied tissues, when comparing the PET-derived result before and following three days of treadmill running. This was contradicted by a significant increase in gene expression of COL1A1 and COL3A1 in skeletal muscle and a strong tendency of enhanced collagen related gene expression in Achilles tendon. Therefore the lack of increase in PET signaling after exercise questions the usability of *cis*-Fpro for detecting exercise-induced changes in collagen synthesis in these tissues. Gene expression of COL1A1 and COL3A1 was not investigated in bone, and very little knowledge exists about the capability of acute or short-term exercise to actually induce bone collagen expression [9], [26]. However, long-term exercise regimes are known to increase bone mass density and based on this, it seems reasonable to expect enhancement of collagen synthesis as a result of exercise.

An obvious reason for *cis*-Fpro not being able to detect the expected changes is that the PET-based method might not be sensitive enough to detect minor changes in collagen synthesis induced by acute exercise. In earlier studies successfully demonstrating exercise-induced collagen synthesis, the isotope-incorporation (^3^H-proline, ^13^C-proline or ^15^N-proline) have been measured only in the acid-precipitated protein fraction and in fact most studies base their investigation on isotope-labeling of hydroxyproline, thereby ensuring the right target, since hydroxylation of proline is specific to collagen molecules [1], [6], [8]. Accordingly, measuring changes in *cis*-FPro uptake *in vivo* in intact tissues with PET might hide (by dilution) small changes in *cis*-Fpro into the collagen fraction. It is possible to quantitate changes in tracer uptake in different metabolic pools using kinetic metabolic compartment modeling with dynamic scans and information on tracer time activity curves and analysis of possible radioactive metabolites in the arterial blood pool (“input function”) by blood drawn from arterial catheters or microdialysis [27]-[29]. However, these methods are technical demanding, especially in small animals and for at least some other PET-labelled amino acids there seems to be a good correlation between SUV and the quantitative methods [30].

Until now the PET-labeled amino acids have mostly been applied to measure protein synthesis in tumors or in cerebrum and only few studies have applied the method to study protein synthesis in skeletal muscle. L-[methyl-11C] methionine has been used in rats [31], dogs [32] and humans [33] for assessment of skeletal muscle protein synthesis. Based on kinetic modeling, the PET-derived protein synthesis rate was comparable to simultaneously conventional stable isotope measurements [32]. Importantly, these studies measured protein synthesis only in resting situations. In a recent study PET in combination with the amino acid analogue [11C]methylaminoisobutyrate were sensitive enough to demonstrate an enhanced amino acid transport in human skeletal muscle during euglycaemic hyperinsulinaemic clamping [34]. However, it should be kept in mind that the changes following exercise are potentially smaller compared to changes evoked by hyperinsulinaemia [35] or even disease-related changes such as protein synthesis in myopathies, tendon rupture or following immobilization, which is known to increase both protein/collagen breakdown and synthesis to a greater extent compared to exercise-induced changes alone [36]. Accordingly, our data do not rule out that *cis*-Fpro may be used in such situations.

Another viable explanation is that the tracer *cis*-Fpro possibly due to the addition of the 18-fluoride to the native proline induces some molecular alterations, which potentially makes the molecule inappropriate as a substrate for collagen synthesis. Reports in the literature have been conflicting regarding this issue, however failure of collagen fibril assembly and inability of fibroblast and cartilage cells to extrude collagen fibers with incorporated fluroro-proline have been reported [12], [37]–[38]. In the present study we were not able to obtain values for tissue protein incorporation comparable to that of Wester et al. [12], where protein incorporation was in the range of 70% in osteosarcoma 240 minutes post-injection. Obviously, it is possible that protein incorporation is more efficient and prompt in fast-growing cancer tissue. In addition there could be differences in tissue acceptability of the *cis*-Fpro or even species differences (mice vs. rats).

The exercise protocol in the present study was similar to an earlier study where PET and the tracer 3′-deoxy-3′-[^18^F]fluoro-thymidine (FLT) were used for detecting an increased cellular proliferation in Achilles tendon and skeletal muscle [39]. Therefore, a parallel increased production of extracellular matrix proteins like the collagens was obviously expected as part of the adaptation process. Despite this, it is still possible that unchanged *cis*-Fpro uptake reflects an actual constant level of musculoskeletal collagen synthesis following three days of running exercise. Endurance training is known to be a less potent anabolic stimulus compared to strength training and other studies have also failed in finding exercise-induced changes in protein and collagen synthesis with the use of tracer methods [8]. Obviously, the increase in gene expression of COL1A1 and COL3A1 in muscle and the tendon speaks against a constant level of collagen synthesis, but theoretically changes in gene expression precedes that of the protein synthesis and therefore is only indicative of actual synthesis at the protein level. A possible way to pursue this issue would be to compare *cis*-Fpro uptake to collagen content in the sampled tissue at the protein level using eg. histology or Western blot.

### Conclusion

The present study investigated the possibility of using PET and *cis*-Fpro as a non-invasive method for detecting collagen synthesis in connective tissues in relation to mechanical loading. The PET images revealed clearly, visible uptake of *cis*-Fpro in the investigated musculoskeletal tissues and the tissue-specific differences with the highest basal uptake in trabecular bone seem to be in accordance with recently published data [1], [22]–[24]. However, since collagen synthesis is a protracted process it might be worthwhile labeling proline with radionuclides with longer halflife, such as ^64^Cu. The uptake of *cis*-Fpro was not changed following three days of treadmill running, which was somewhat in conflict with the simultaneous increased COL1A1 and COL3A1 gene expression in skeletal muscle and Achilles tendon. *cis*-Fpro as used in the present study appears questionable as a tracer for assessment of collagen formation during adaptation to exercise. However, further studies with greater mechanical loading of the tissues and possible also applying a kinetic compartment model, which likely can increase the sensitivity of the method, must be performed before excluding *cis*-Fpro as an appropriate method for non invasive *in-vivo* assessment of exercise-induced changes in musculoskeletal collagen synthesis.
